# Effect of *CCL5* expression in the recruitment of immune cells in triple negative breast cancer

**DOI:** 10.1038/s41598-018-23099-7

**Published:** 2018-03-20

**Authors:** Jhajaira M. Araujo, Andrea C. Gomez, Alfredo Aguilar, Roberto Salgado, Justin M. Balko, Leny Bravo, Franco Doimi, Denisse Bretel, Zaida Morante, Claudio Flores, Henry L. Gomez, Joseph A. Pinto

**Affiliations:** 1 Unidad de Investigación Básica y Traslacional, Oncosalud-AUNA, Lima, Peru; 2 Escuela de Ingeniería Biotecnológica, Universidad Católica de Santa María, Arequipa, Peru; 3 Breast Cancer Translational Research Laboratory, Institut Jules Bordet, Brussels, Belgium; 4 Department of Medicine, Vanderbilt-Ingram Comprehensive Cancer Center, Vanderbilt University, Nashville, Tennessee USA; 5 Escuela de Medicina Humana, Universidad Privada San Juan Bautista, Av. José Antonio Lavalle s/n Hacienda Villa, Chorrillos, Lima 09, Peru; 6 Grupo de Estudios Clínicos Peruano Oncológico (GECOPERU), Calle Tinajones 177, Surco, Lima, Peru; 7 Departamento de Oncología Médica, Instituto Nacional de Enfermedades Neoplasicas, Lima, Peru

## Abstract

Triple negative breast cancer (TNBC) is the most aggressive form of breast cancer with limited options of targeted therapy. Recent findings suggest that the clinical course of TNBC may be modified by the presence of tumor-infiltrating lymphocytes (TILs) and chemokine’s expression, such as *CCL5*. Diverse studies have shown that *CCL5* suppresses anti-tumor immunity and it has been related to poor outcome in different types of cancer while in other studies, this gene has been related with a better outcome. We sought to determine the association of *CCL5* with the recruitment of TILs and other immune cells. With this aim we evaluated a retrospective cohort of 72 TNBC patients as well as publicly available datasets. TILs were correlated with residual tumor size after neoadjuvant chemotherapy (NAC) and *CCL5* expression. In univariate analysis, TILs and *CCL5* were both associated to the distant recurrence free survival; however, in a multivariate analysis, TILs was the only significant marker (HR = 0.336; 95%IC: 0.150–0.753; P = 0.008). CIBERSORT analysis suggested that a high *CCL5* expression was associated with recruitment of CD8 T cells, CD4 activated T cells, NK activated cells and macrophages M1. The *CD8A* gene (encoding for CD8) was associated with an improved outcome in several public breast cancer datasets.

## Introduction

Triple-negative breast cancer (TNBC) is a heterogeneous group of breast tumors characterized by the lack of expression of estrogen receptor, progesterone receptor and HER2. TNBC is the most aggressive subtype of breast tumors because its biology and the limited options of targeted therapy^1,2^. Several efforts are being conducted to characterize its complexity and heterogeneity by combining structural and functional genomics approaches^3–6^.

Nowadays, there are several reports demonstrating the crucial role of immunity in TNBC biology, suggesting the potential involvement of immunotherapy to treat this malignancy where tumor infiltrating lymphocytes (TILs) are associated with better outcomes and response to chemotherapy^7–12^. TILs are constituting an important factor to predict in the outcome of TNBC in the neoadjuvant (pretreated or treated tumors) or in the adjuvant setting^10,13–15^. Evaluate TILs is a raw measurement of an immunological process where information of cellular subsets or cellular states is missing. Higher expression of cytotoxic molecules, T cell-related genes, Th1-related cytokines, and B cell markers were previously correlated with pathological complete response in breast cancer treated with anthracycline-based NAC^8,11,13^.

Despite the number of covariates that could influence biologically the activity of infiltrating lymphocytes, TILs evaluation *per se* has shown to predict the clinical outcome independently of other prognostic factors. Interestingly, a recent work has shown that some gene regulatory networks are shared among different immune cell subtypes while local sub networks define the phenotype; however, tumor-induced changes in local sub networks confers plasticity to immune cells producing a tumor-friendly environment, suggesting a need to improve the molecular characterizations of infiltrating immune cells^16^.

In a previous work to identify genes of prognostic value in TNBC, we identified *CCL5*, *DDIT4* y *POLR1C* as independent prognostic factors where a high *CCL5* expression was associated with a better prognosis (HR = 0.6, CI95%: 0.53–0.86)^17^. This good prognostic value was contrasting with many reports evaluating other cancer types. The scientific literature describe a dual role for *CCL5* in cancer, attributing it a good outcome or a poor outcome role^18,19^.

The *CCL5* (*C-C motif chemokine ligand 5*) gene belongs to the chemokine superfamily and encodes a protein that induces lymphocytes and monocytes migration. This gene has a higher expression in HER2-enriched and basal breast cancer subtypes than luminal tumors^20,21^. There are reports indicating that CCL5 attracts immunosuppressive cells promoting the immune tolerance or conversely, it is involved in the recruitment of immune effectors cells^22^.

We evaluated the association of *CCL5* expression with clinicopathological features and the recruitment of TILs and subsets of immune cells in TNBC, and its influence in patients’ outcome.

## Results

### Clinicopathological features according to CCL5 expression and TILs

In 72 TNBC evaluated patients, the median age was 47.5 years (range: 24 to 78). There were not statistical differences in the clinopathological features, except in the residual tumor size with larger tumors in low TILs group (P = 0.017) and *CCL5* (P = 0.053) **(**Table 1**)**.

**Table 1:** Clinicopathological characteristics of evaluated patients according to *CCL5* expression and TILs count.

Clinicopathological characteristics	*CCL5*		P-value	TIL’s count		P-value
	<median n(%)	≥median n(%)		Low TILs	High TILs	
				n (%)	n (%)	
**TOTAL**	36 (50.0)	36 (50.0)		43 (59.7)	29 (40.3)	
**Age**			0.281			0.322
Median (range)	44.5 (24–78)	48.5 (29–72)		46.9 (24–72)	49.8 (29–78)	
**Menopausal Status**			0.238			0.812
Pre	20 (58.8)	14 (41.2)		21 (61.8)	13 (38.2)	
Post	16 (42.1)	22 (57.9)		22 (57.9)	16 (42.1)	
**Clinical stage**			0.674			0.679
IIA-IIB	2 (33.3)	4 (66.7)		3 (50.0)	3 (50.0)	
IIIA-IIIC	34 (51.5)	32 (48.5)		40 (60.6)	26 (39.4)	
**Chemotherapy**			NA			NA
A	11 (34.4)	21 (65.6)		17 (53.1)	15 (46.9)	
A + T	25 (67.6)	12 (32.4)		25 (67.6)	12 (32.4)	
Others	0 (0)	3 (100)		1 (33.3)	2 (66.7)	
**Node Involvement**			0.322			0.211
Negative	10 (41.7)	14 (58.3)		12 (50.0)	12 (50.0)	
Positive	26 (55.3)	21 (44.7)		31 (66.0)	16 (34.0)	
**Positive Nodes**			0.312			0.409
0	10 (41.7)	14 (58.3)		12(50.0)	12 (50.0)	
1–3	12 (48.0)	13 (52.0)		17 (68.0)	8 (32.0)	
>3	14 (63.6)	8 (36.4)		14 (63.6)	8 (36.4)	
**Residual tumor size** (**mm)**			0.053			**0**.**017**
Median (range)	50 (0–250)	34.5 (0–125)		50 (0–250)	34.5 (0–80)	

^*^NA: not applicable.

### There is a direct correlation between *CCL5* and TILs

**Figure 1:**
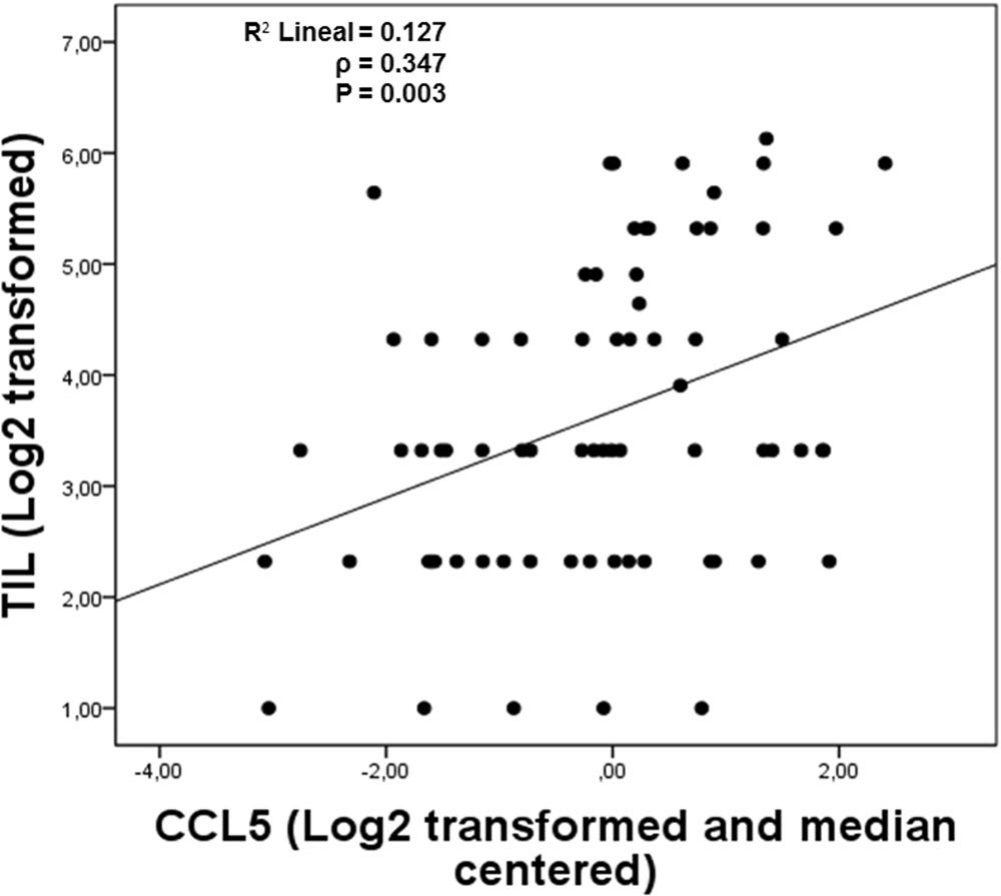
A positive correlation between CCL5 and TILs count was observed in the Peruvian cohort (P = 0.003).

**Figure 2:**
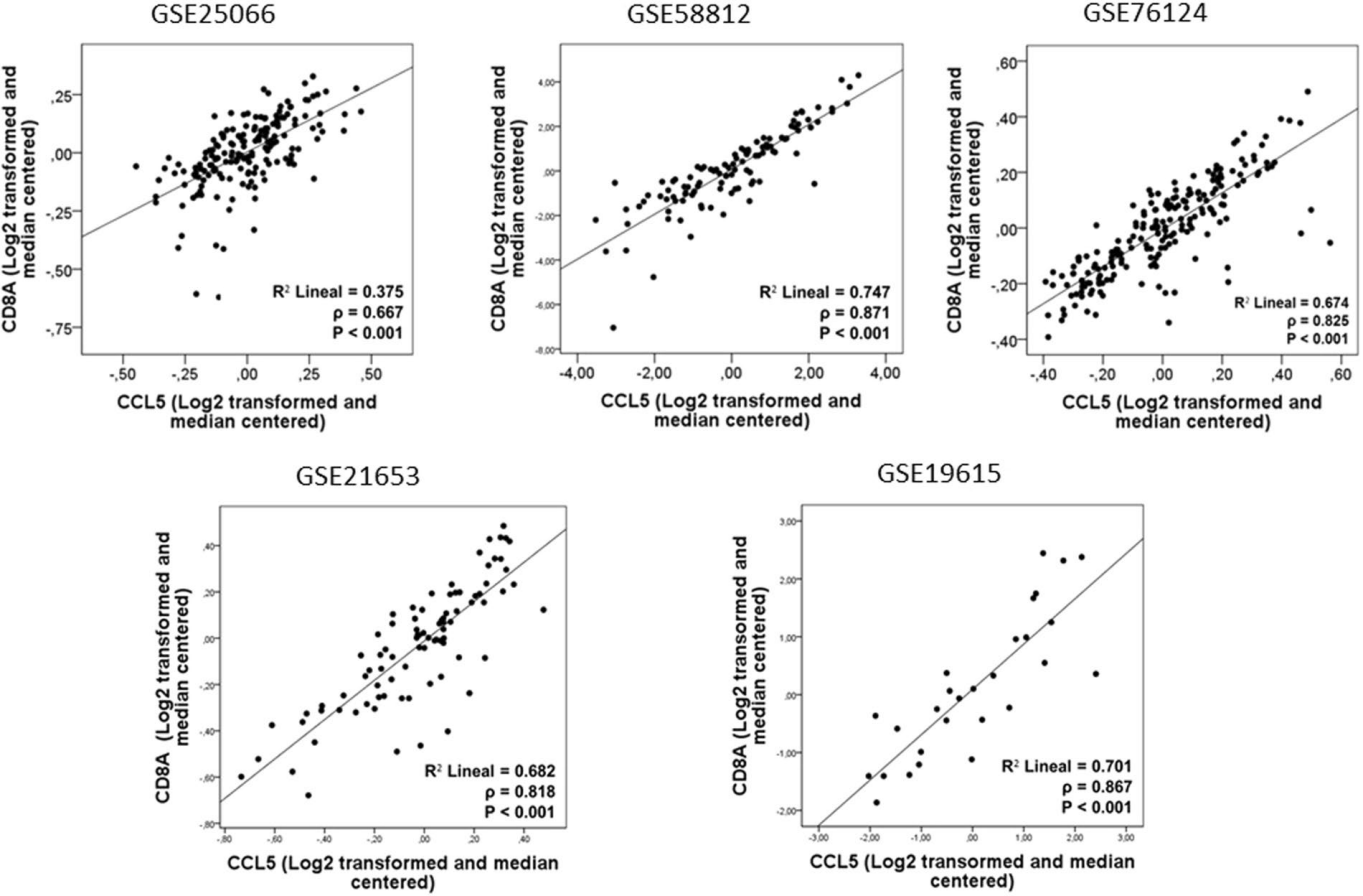
Expression of *CCL5* was directly correlated with the expression of *CD8A* in all datasets.

**Figure 3:**
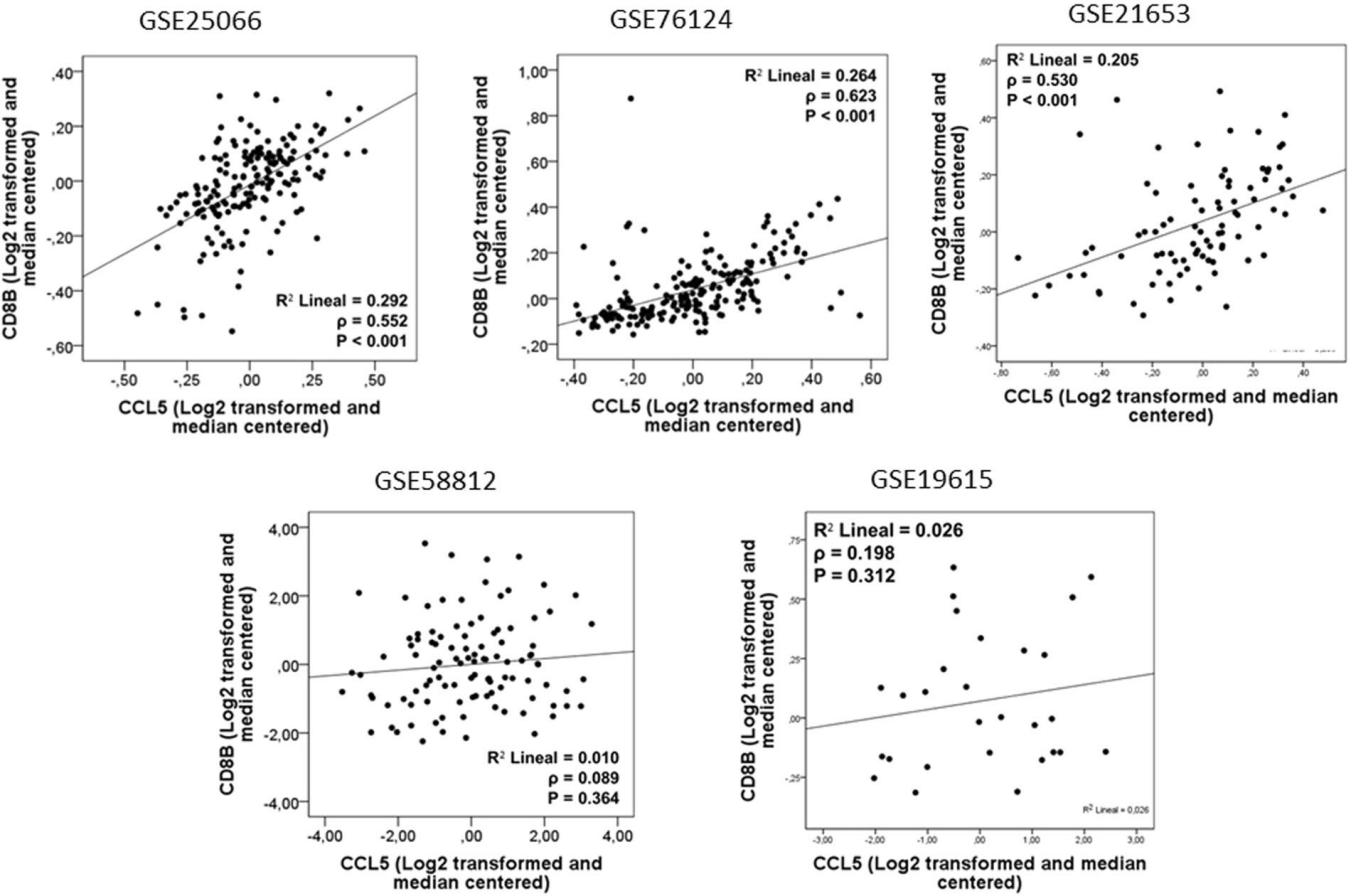
*CD8B* expression was associated with CCL5 in 3 out 5 datasets of TNBC.

A significant correlation between TILs and *CCL5* (ρ = 0.347, P = 0.003) was observed in our retrospective TNBC cohort **(**Fig. 1**)**. In addition, in independent public datasets, a positive correlation between *CCL5* and *CD8A* was observed (GSE25066: ρ = 0.667, P < 0.001; GSE58812: ρ = 0.871, P < 0.001; GSE76124: ρ = 0.825, P < 0.001; GSE21653: ρ = 0.818, P < 0.001; GSE19615: ρ = 0.867, P < 0.001) **(**Fig. 2**)**, as well as a correlation between *CCL5* and *CD8B* (GSE25066: ρ = 0.552, P < 0.001; GSE76124: ρ = 0.623, P < 0.001; GSE21653: ρ = 0.530, P < 0.001) **(**Fig. 3**)**.

### TILs and *CCL5* are related with the outcome

In the univariate analysis of the retrospective cohort for distant metastases-free survival, TILs count (HR = 0.276; 95%IC: 0.128–0.593; P = 0.001) and *CCL5* (HR = 0.401; 95%IC: 0.206–0.781; P = 0.007) were both associated with distant recurrence free survival (DRFS). In the multivariate analysis between *CCL5* and TILs, TILs remains as an independent prognostic factor (HR = 0.336 per unit of change; 95%CI: 0.150–0.753; P = 0.008) while *CCL5* expression had not significant prognostic value (HR = 0.573 per unit of change; 95%CI: 0.285–1.154; P = 0.119) **(**Table 2**)**. Due to tumor heterogeneity could add bias in the evaluation of biomarkers we performed 1000 resampling with the bootstrap method to verify the robustness of the model^23^. After this analysis, results obtained were a HR = 0.37 (95%CI: 0.135–0.827) and HR = 0.56 (95%IC: 0.239–1.240) for TILS and CCL5, respectively.

**Table 2.** Univariate and multivariate analysis of TILs count and *CCL5* expression as categorical variables.

	HR	CI95%	P-value
Univariate analysis			
TILs			
Low	1		
High	0.276	0.128–0.593	0.001
CCL5			
<median	1		
≥median	0.401	0.206–0.781	0.007
**Multivariate analysis**			
TILs			
Low	1		
High	0.336	0.150–0.753	0.008
CCL5			
<median	1		
≥median	0.573	0.285–1.154	0.119

**Figure 4:**
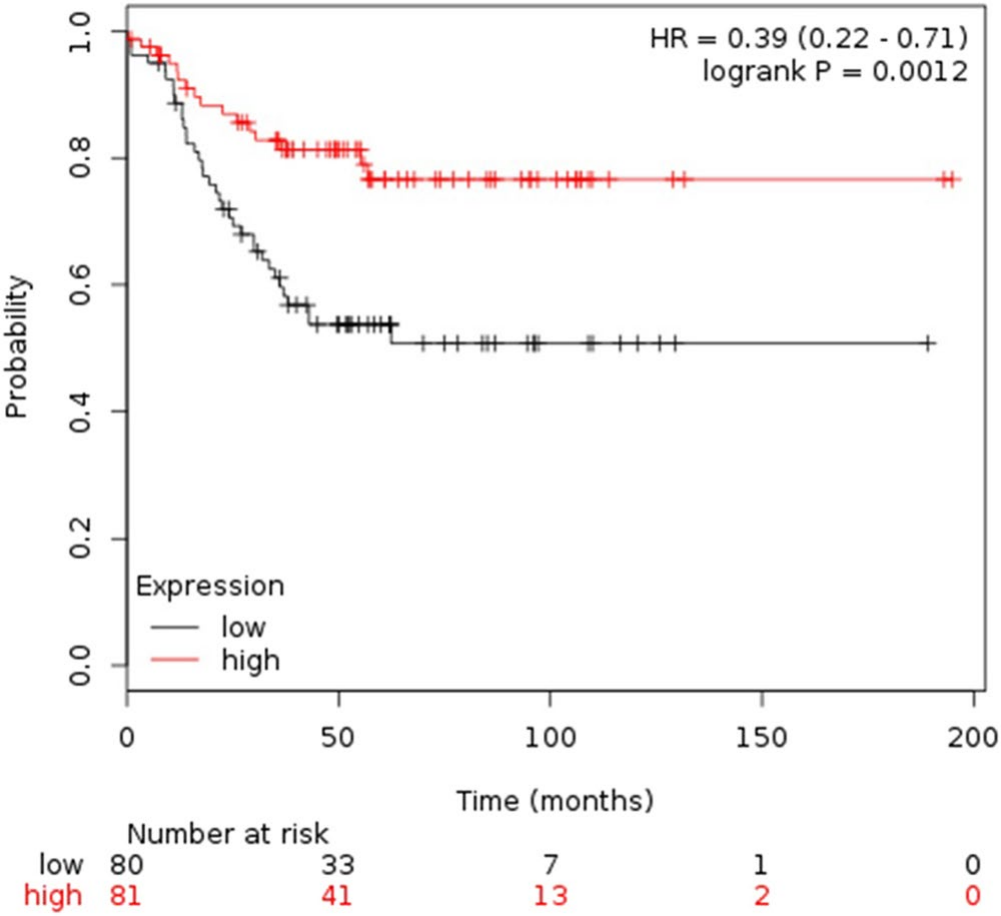
Meta-analysis of *CCL5* in recurrence-free survival (RFS) in TNBC (using the median of expression as cutoff) in databases of KM plotter. A High expression of CCL5 was associated with good prognosis (P = 0.0012).

An analysis of *CCL5* expression in KM plotter (http://kmplot.com/)^24^ shown that a high *CCL5* expression was associated with a better outcome in TNBC patients, either in the meta-analysis of all datasets (HR = 0.39, CI95%: 0.22–0.71; P = 0.0012) **(**Fig. 4**)** or analyzing each dataset separately (n = 3) (Supplementary Figure 1).

### Immune cell composition according to *CCL5* expression

**Figure 5:**
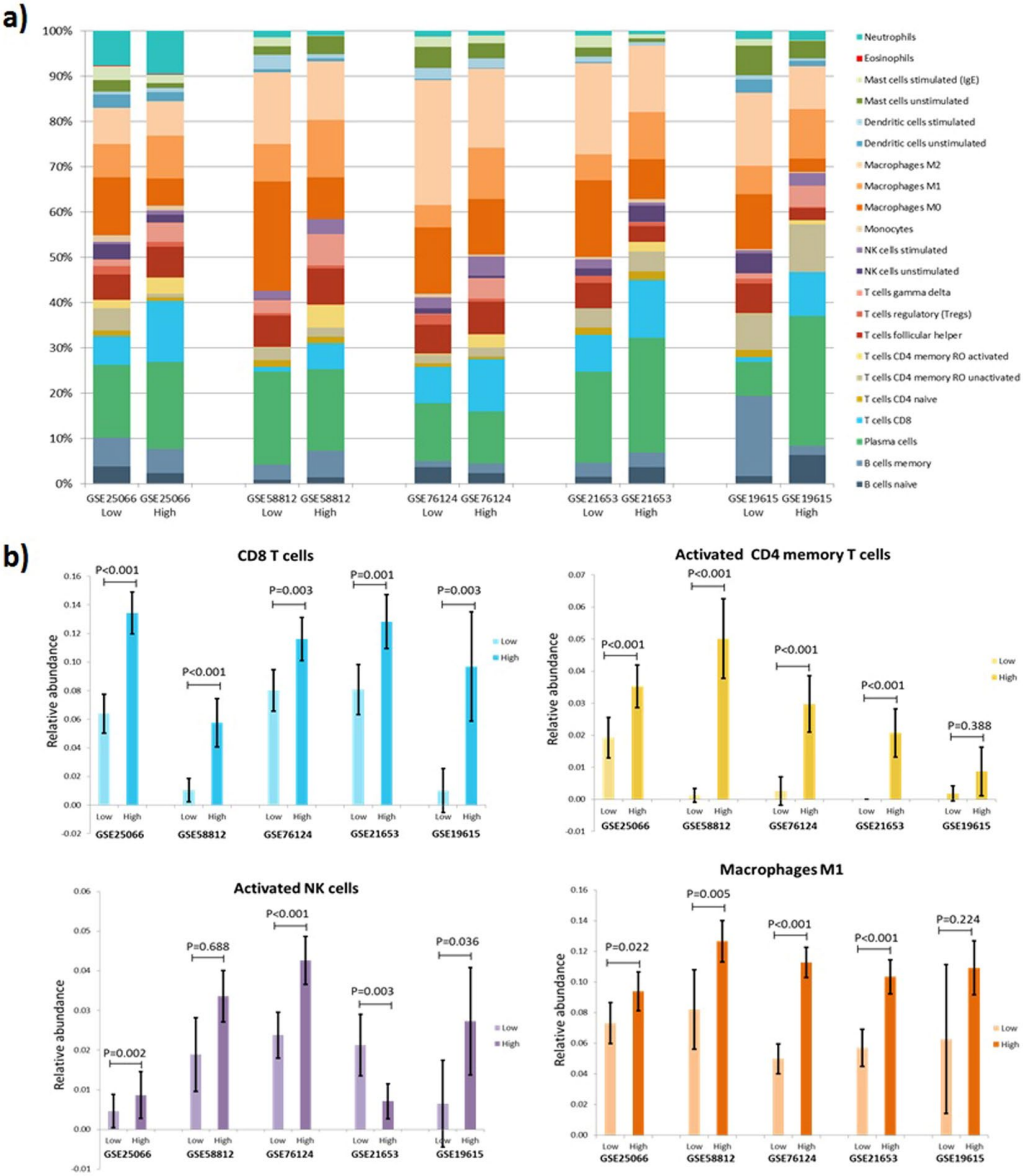
Relative fractions of 22 leukocyte subtypes (LM22 signature) evaluated by CIBERSORT in five TNBC datasets according to *CCL5* expression (1^st^ tertile vs 3^rd^ tertile) **(a)**. Differences between immune cell subtypes according to *CCL5* expression. Analysis was limited to cases with CIBERSORT p-value < 0.05 **(b)**.

CIBERSORT analysis (https://cibersort.stanford.edu/)^25^ in five public TNBC datasets suggested that a high *CCL5* expression (comparing the upper tertile vs the lower tertile) is associated with recruitment of CD8 cells, activated CD4 memory T cells, activated NK cells and Macrophages M1 **(**Fig. 5**)**. Regarding regulatory T cells (Treg) cells, an increase was observed in patients with low expression of *CCL5*, but this was statistically significant in only two datasets (GSE25066: 2% vs. 1%, P = 0.029; GSE76124: 2% vs. 1%, P < 0.001). Similar results were found when datasets were split into two (median as cutoff) or four groups (upper quartile vs. lower quartile) **(**Supplementary data S1**)**. All relative fractions and P-values obtained from the CIBERSORT analysis are showed in Supplementary data S2.

### *CD8A* expression is related with the outcome

**Figure 6:**
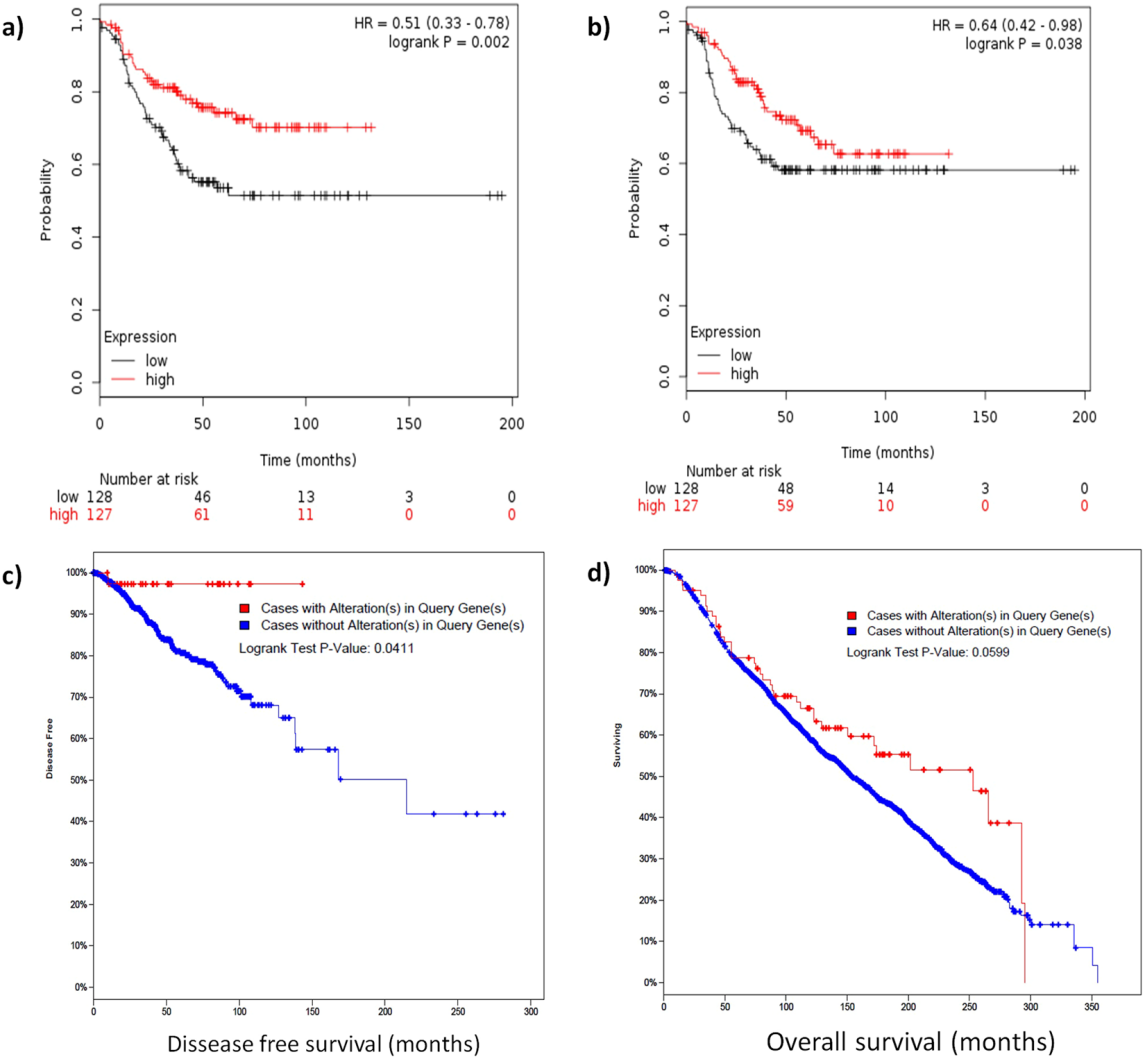
Meta-analysis in KM-Plotter showed that an high expression of CD8A **(a)** and CD8B **(b)** are related with a better relapse free survival in TNBC. CD8A overexpression is related with better disease free survival in the TCGA **(c)** and better overall survival in the METABRIC **(d)** datasets (all breast cancer subtypes).

Because the main lineage biomarkers in CD8 T cells are *CD8A* and *CD8B* expression, these genes were used as indicators of CD8 cells infiltration. A high expression of either *CD8A* or *CD8B* was related with an improved outcome in KM-Plotter analysis **(**Figs 6A,B**)**. Over expression of *CD8A* was related with a better survival in the TCGA and METABRIC datasets **(**Figs 6C and 3D**)**.

## Discussion

In this study we combine analysis of patients’ samples and evaluation in bioinformatics platforms to assess the effect of *CCL5* in the infiltration of immune cells. Although we inferred the immune cell composition form genomic data, we used a robust and validated algorithm^26^.

Several studies have pointed the value of TILs in the outcome in breast cancer and other solid tumors. A previous study showed that a 20% cutoff in stromal TILs is able to discriminate low vs high infiltration and detect significant differences in the outcome in TNBC patients^27^. In a meta-analysis of 8 studies, Ibrahim *et al*. (2014), showed that triple negative breast tumors rich in TILs had an 30% reduction in risk of recurrence, 22% reduction in risk of distant recurrence and 34% in reduction in the risk of death^28^.

In our analysis, there was direct correlation between TILs and CCL5 expression in TNBC; therefore other reports describe that not only pro-immune markers (CCL5 [ρ = 0.677, P < 0.001], CD45RO, CD80, CXCL9, and CXCL13), but also immunosuppressive markers such as LAG3, IDO1, CTLA-4, TIGIT, BTLA, and FOXP3 had a positive correlation with increased TILs^15,29^.

In regard to *CCL5*, several reports describe that a high expression of this gene is associated to a poor outcome^30,31^. There are several mechanisms possibly linking *CCL5* with aggressiveness and oncogenic features. Mesenchymal stem cells are induced by tumor cells to secret CCL5 to enhance motility and metastasis^32^. Interestingly, in a model of gastric cancer, tumor cells induced CD4+ T-cells to secrete CCL5, which in turn, induced apoptosis in CD8+ T cells, while neutralization of CCL5 with monoclonal antibodies induced tumor suppression^33^. After radiotherapy in non-small cell lung cancer, *CCL5* is overexpressed and can induce macrophage infiltration, promoting tumor progression^34^. Regarding breast cancer, it has been described that *CCL5*-deficient mice are resistant to mammary tumor growth^19^. In stage II breast cancer, high expression of *CCL5* (assessed by immunohistochemistry) has been associated with disease progression^35^.

On the other hand, in melanoma, intratumoral injection of IFN-β induces expression of CCL5 and CXCR3 ligands and administration of IFN-β with anti-PD-1 monoclonal antibodies suppressed the tumor growth and prolonged the survival in a murine model^36^.

In ER- breast cancer, tumor-infiltrating FOXP3+ lymphocytes and *CCL5* expression were associated to a good outcome^37–39^. deLeeuw *et al*. (2012) showed that the role in the outcome of FOXP3+ tumor-infiltrating lymphocytes depends of the tumor site and microenvironment features^40^. In that way, these conditions could be the responsible of the different prognostic value seen in *CCL5*.

In our study, *CCL5* lacks of prognostic ability when is adjusted to TILs **(**Table 2**)** suggesting that in TNBC, levels of TILs infiltration is the most important immunological variable. In a similar way, Denkert *et al*. (2015) reported that *CCL5* is also related to an increased pCR in TNBC patients (OR, 1.30 per ≥ Δ Ct; 95%CI, 1.07 to 1.56; P = 0.007), but after adjusting to TILs count it was no significant^41^.

We identified several patterns of infiltrations associated to high *CCL5* expression characterized by a higher infiltration of CD8 T-cells, CD4 memory activated T-cells, NK activated T-cells and Macrophages M1 (analyzed in CIBERSORT). Because the higher infiltration of CD8 T-cells we next evaluated the association of *CD8A* with the outcome in KM-plotter (for TNBC) and in two genomic projects, TCGA and METABRIC (for all subtypes), where this gene was associated to a better outcome **(**Fig. 3**)**. The mechanistic antitumor role of CD8 T-cells, CD4 memory activated T cells should be studied in detail.

Traits of infiltrating immune sets were previously correlated with the clinical outcome. Interestingly, the prognostic value of immune cells is maintained among different cancer types^26^. In the particular case of breast cancer, a prior study described that tumor infiltration with B cells memory, monocytes and dendritic cell resting were associated to resistance to NAC and macrophages M1 and B cells naïve were related to the pathological complete response in ER positive tumors while in ER negative tumors, macrophages M2 and mast cell resting were related to resistance to NAC and T-cells follicular helper were associated with higher probability of pathological complete response^42^^.^

In conclusion, although *CCL5* expression is associated to a better outcome in breast cancer, particularly in TNBC, TILs assessment remains the stronger and more significant prognostic immunological marker although characterization of cellular states of TILs should provide a more precise prognostic biomarker.

## Material and Methods

### Patients

We evaluated a retrospective cohort of Peruvian patients who had residual tumors after NAC whose tumors were evaluable for TILs and *CCL5*. In total, 72 patients were included in the analysis (one patient was excluded because its TILs count was zero). The clinicopathological parameters evaluated were: age at diagnosis, menopausal status, clinical stage, node involvement, residual tumor size, TILs count, distant recurrence status and time to distant recurrence.

### Breast Cancer Datasets

Five independent datasets were obtained from GEO (https://www.ncbi.nlm.nih.gov/geo/), to evaluate the immune cells composition according to the expression of *CCL5*.

#### GSE25066

We selected 178 TNBC cases (determined by immunohistochemistry). Samples were collected before NAC. Gene expression profiling was measured with U133A Affymetrix microarray platform (Affymetrix, Santa Clara, CA, USA).

#### GSE58812

We evaluated all the 107 TNBC of this dataset. Gene expression was profiled with Affymetrix Human Genome U133 Plus 2.0 Array (Affymetrix, Santa Clara, CA, USA).

#### GSE76124

This dataset was composed of 198 TNBC cases. Gene expression was profiled with Affymetrix Human Genome U133 Plus 2.0 Array (Affymetrix, Santa Clara, CA, USA).

#### GSE21653

We included 87 TNBC cases. Gene expression was profiled with Affymetrix Human Genome U133 Plus 2.0 Array (Affymetrix, Santa Clara, CA, USA).

#### GSE19615

We evaluated 28 TNBC cases. Gene expression was profiled with Affymetrix Human Genome U133 Plus 2.0 Array (Affymetrix, Santa Clara, CA, USA).

### TILs assessment

Post-NAC tumors were submitted for pathologic evaluation. After they were stained with H&E staining, determination of percentage of stromal lymphocytic infiltration (%TIL) was done according to method described by Dieci *et al*.^43^ A cutoff value of 20% was selected to discriminate high vs low TILs^27^.

### Gene expression analysis

Tumor-rich regions of formalin-fixed paraffin embedded tumor blocks were serially cuttted in 3–6 μm sections. RNA was extracted and purified using the RNeasy FFPE Kits (Qiagen). Gene expression analysis was performed by NanoString (Seattle, WA). Samples were assayed on a Bioanalyzer (Agilent, Santa Clara,CA) to determine the concentration of RNA. Raw data was subtracted from background with spike-controls and then was normalized by dividing the geometric mean of seven housekeeper-control genes: ACTB, B2M, G6PD, GAPDH, GUSB, POLR1B, RPLPO and TUBB.

### Evaluation of *CCL5* and TILs correlation

Housekeeper-normalized gene expression values of *CCL5*, *CD8A* and *CD8B* were log2 transformed and median centered, while TILs count was log2 transformed. Spearman’s rank correlation analysis was used to assess the relationship between these markers.

### Survival Analysis

In the Peruvian cohort, cox Proportional-Hazards Regression was used to evaluate the impact of TILs and *CCL5* in the outcome. Both were evaluated as categorical variables (TILs < 20% and TILs ≥ 20%; *CCL5* < median and *CCL5* ≥ median). To validate the result of the cox model, the HR and 95% confidence intervals were estimated with 1,000 bootstrap resampling. The analysis was done using the package boot in R language.

Additionally, the effect of *CCL5* on recurrence free survival was assessed using the online tool KM plotter (http://kmplot.com/analysis/)^24^ in all TNBC patients (median as cutoff).

### Analysis of immune cells composition from gene expression data

The datasets were independently analyzed. The probe’s IDs were changed to its respective genes symbols and then genes expressions levels were collapsed to the maximum value. Each data set was split according to *CCL5* expression using tertiles where samples with central values (group 2) were excluded.

We used the online analytical platform CIBERSORT (https://cibersort.stanford.edu/)^25^ in order to estimate the relative proportions of 22 immune cell types. Analyses were done with 100 permutations, enabled quantile normalization and default statistical parameters. The results were filtered by a maximum p-value of 0.05. Comparisons of relative fractions were done with the Mann–Whitney U test.

### Evaluation of *CD8* effect on the outcome

We assessed the impact of *CD8A* and *CD8B* expression on the outcome. For this analysis we used two online platforms; KM plotter (http://kmplot.com/analysis/)^24^ (TNBC cases) and cBioPortal (http://www.cbioportal.org/)^44,45^ (TCGA provisional and METABRIC datasets).

### Ethical considerations

This study involves a reanalysis of gene expression and clinical data obtained in previous studies.

## Electronic supplementary material

Supplementary Information

Supplementary data S1

Supplementary data S2
