# Supplementary material for: Effect of CCL5 expression in the recruitment of immune cells in triple negative breast cancer
Source: Sci Rep. 2018 Mar 20;8:4899. doi: 10.1038/s41598-018-23099-7 (PMC5861063; doi:10.1038/s41598-018-23099-7)
Supplement: Supplementary file 1 — Supplementary Information [file 41598_2018_23099_MOESM1_ESM.docx]

**Effect of *CCL5* expression in the recruitment of immune cells in triple negative breast cancer**

Jhajaira M. Araujo^1^, Andrea C. Gomez^2^, Alfredo Aguilar^1^, Roberto Salgado^3^, Justin M. Balko^4^, Leny Bravo^5^, Franco Doimi^1^, Denisse Bretel^1,6^ Zaida Morante^1,7^, Claudio Flores^1^, Henry L. Gomez^1,7^, Joseph A. Pinto^1^

**Affiliations:**

1, Unidad de Investigación Básica y Traslacional, Oncosalud-AUNA, Lima, Peru.

2, Escuela de Ingeniería Biotecnológica, Universidad Católica de Santa María, Arequipa, Peru.

3, Breast Cancer Translational Research Laboratory, Institut Jules Bordet, Brussels, Belgium.

4, Department of Medicine, Vanderbilt-Ingram Comprehensive Cancer Center, Vanderbilt University, Nashville, Tennessee, USA.

5, Escuela de Medicina Humana, Universidad Privada San Juan Bautista, Av. José Antonio Lavalle s/n Hacienda Villa, Chorrillos. Lima 09, Peru

6, Grupo de Estudios Clínicos Peruano Oncológico (GECOPERU), Calle Tinajones 177, Surco, Lima-Peru

7, Departamento de Oncología Médica, Instituto Nacional de Enfermedades Neoplasicas, Lima, Peru.

**Correspondence:**

Henry L. Gómez, MD, PhD

Unidad de Investigación Básica y Traslacional, Oncosalud-AUNA, Lima, Peru Guardia Civil 571, San Borja

Lima 41, Peru

Email: hgomezmoreno@gmail.com

Phone: +511 5137900 Ext. 2231


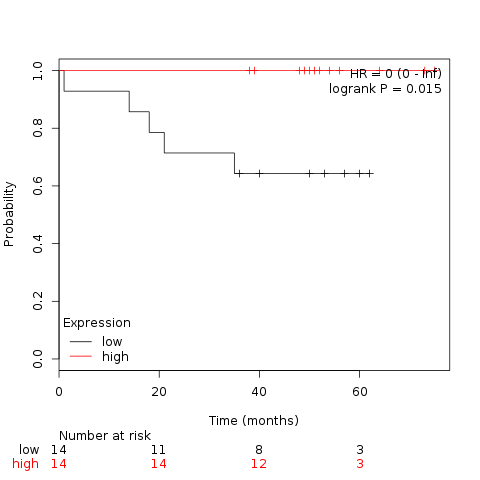


**GSE19615**


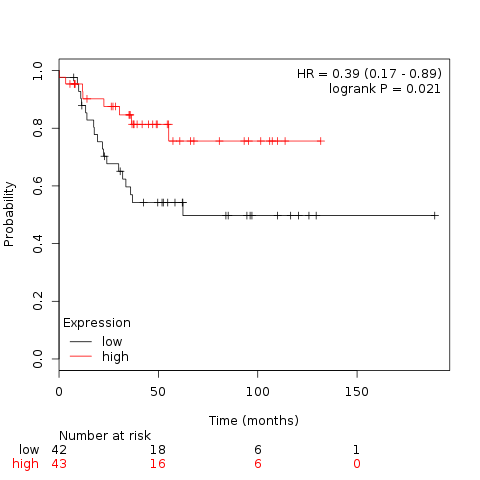


**GSE21653**


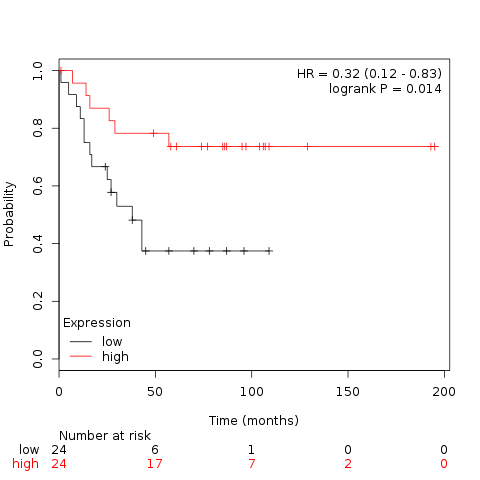


**E-MTAB-365**

**Supplementary Figure 1.-** Analysis of the impact of CCL5 in recurrence-free survival (RFS) in TNBC (using the median of expression as cutoff ) in the databases of KM plotter analyzed separately. A High expression of CCL5 was associated with good prognosis in all datasets (GSE19615, P=0.015; GSE21653, P= 0.021; E-MTAB-365, 0.014)**.**
